# Removal of Cr(VI) by biochar derived via co-pyrolysis of oily sludge and corn stalks

**DOI:** 10.1038/s41598-022-14142-9

**Published:** 2022-06-14

**Authors:** Lei Han, Jinling Li, Tiantian Zhang, Chengtun Qu, Tao Yu, Bo Yang, Zhiguo Shao

**Affiliations:** 1 College of Chemistry and Chemical Engineering, Xi’an Shiyou University, Xi’an, 701165 China; 2 Shaanxi Key Laboratory of Environmental Pollution Control Technology and Reservoir Protection of Oilfield, Xi’an, 701165 China; 3 State Key Laboratory of Petroleum Pollution Control, CNPC Research Institute of Safety and Environmental Technology, Beijing, 102206 China

**Keywords:** Environmental sciences, Energy science and technology

## Abstract

The co-pyrolysis of oily sludge with biomass to prepare carbon materials is not only an effective way to mitigate oily sludge pollution, but it is also a method of obtaining carbon materials. In this study, a carbon material (OS-CS AC) was obtained by the direct co-pyrolysis of oily sludge (OS) and corn stalks (CS) and then applied to Cr(VI) removal. According to the hydroxy and carboxy masking experiments and the characterization of OS-CS AC by FT-IR, SEM, XPS, XRD, and N_2_ physical adsorption–desorption, Cr(VI) can be adsorbed efficiently through pore filling, the surface oxygen-containing functional groups can promote the reduction of Cr(VI) to Cr(III) through electron donors, and the greater the electrostatic attraction between the electron-donating functional groups of OS-CS AC and the Cr(VI) is, the stronger the ability to remove Cr(VI). In addition, the removal process was discussed, and the results indicated that the McKay kinetic model, Langmuir isotherm model and Van't Hoff thermodynamic model were the most suitable models for removal. The main factors affecting the removal of Cr(VI) were discussed, and the removal of Cr(VI) reached 99.14%, which gives a comprehensive utilization way of oily sludge and corn stalks.

## Introduction

With the acceleration of industrial development, the overuse of landfills, and the stacking of domestic garbage, large amounts of heavy metals enter rivers and lakes through sewage systems^1^. These heavy metals have been reported to bioaccumulate and express biotoxic characteristics^2^ since they are easily transported through the biological chain, endanger human health and the ecosystem, and cause large-scale pollution^3^. Chromium (Cr) is a significant industrial material used for production in many industries, such as leather tanning, paint processing, electroplating, textile printing, and battery fabrication^4^. These industries produce large amounts of Cr-containing wastewater. Without proper disposal, these toxic substances severely harm ecosystems and give rise to numerous diseases^5^. Therefore, Cr has been listed as a priority pollutant in many countries. Cr(III) and Cr(VI), which have different mobilities and carcinogenicities, are the dominant forms of Cr in aqueous solutions^6^. Owing to its strong solubility, mobility, and toxicity, Cr(VI) has extremely teratogenic and carcinogenic effects on humans and animals^7^. Therefore, it is essential to control the concentration of Cr(VI) in wastewater to protect ecosystems and human health.

In recent years, many technologies (e.g., ion exchange, photocatalysis, membrane, adsorption, and chemical reduction) have been developed to remove Cr(VI) from wastewater^8^. Among these technologies, adsorption is more attractive for Cr(VI) removal because of its high efficiency and simple operation^9^. However, the unclear removal mechanism and the high cost of modification limit its application in large-scale production. To solve these problems, some research has reported that the mechanism of Cr(VI) removal includes electrostatic attraction, reduction of Cr(VI) and adsorption of Cr(VI)^10,11^. Zhu et al. used a haematite nanoparticle-modified biochar-clay granular composite to explore the relationship between the change in zeta potential and Cr(VI) removal ability at different pH values and revealed the influence of electrostatic attraction on the adsorption process^12^. Chen et al. used modified biochar to study the relationship between the pHpzc (point of zero charge) and the ability to remove Cr(VI) at different pH values and revealed the influence of electrostatic attraction on the adsorption process^13^. Both of these results show that the ability to remove Cr(VI) increased with increasing electrostatic attraction, even at different pH values. However, the reduction ability and adsorption ability of carbon materials for Cr(VI) removal may change when the pH differs, further influencing electrostatic attraction. Therefore, it is necessary to explore the influence of electrostatic attraction on the Cr(VI) removal ability without the effect of pH.

Oily sludge is an inevitable byproduct of the petrochemical industry during the production, collection, and transportation of petrochemicals. In China, more than one million tons of oily sludge are produced every year^14^. Currently, the main disposal methods for oily sludge include stacking, landfilling, and incineration^15^. However, these methods have been moderately limited or prohibited because of the risks of heavy metal pollution, especially Cr pollution^16^. Some studies have shown that pollutants such as heavy metals in oily sludge can be transformed to a more stable state via pyrolysis^17^. Thus, converting oily sludge to carbon materials through pyrolysis could be an interesting alternative to treating oily sludge, considering that it eliminates heavy metal pollution and converts oily sludge into a usable resource^18^. However, the adsorption capacities of the prepared activated carbon have not been satisfactory in applications because of the oily sludge, which has a relatively low carbon content and high ash content^19^. In recent years, many researchers have prepared adsorption materials by co-pyrolyzing sludge with biomass such as cotton stalks^20^ and bagasse^21^ and have explored the potential application prospects of the obtained carbon materials. The abovementioned studies showed that the addition of biomass to oily sludge during pyrolysis could improve the properties of the adsorption materials.

China is one of the world’s largest corn producers. In recent decades, the unsuitable disposal of corn stalks (e.g., burning and landfilling) has caused inevitable environmental problems. However, because of their high fixed carbon content, low ash content, and extensive sources, corn stalks have high use value^22^. Previous studies have shown that the addition of corn stalk could increase the surface area and pore structure of carbon materials^23^, which can improve the adsorption active sites and adsorption capacity of carbon materials for heavy metals. However, to the best of our knowledge, there has been no study on the application of oily sludge co-pyrolysis with corn stalks to prepare carbon materials for Cr removal, and no possible removal mechanism has been proposed. Therefore, the co-pyrolysis of oily sludge and corn stalks to prepare carbon materials may accomplish their synergistic utilization.

In this study, carbon materials (OS-CS AC) were prepared by co-pyrolysis using oily sludge (OS) and corn stalks (CS) as raw materials and applied to remove Cr(VI) from wastewater. The effect of electrostatic attraction between the electron-donating functional groups of OS-CS AC and Cr(VI) on the removal of Cr(VI) was explored by carboxyl and hydroxyl masking experiments^24,25^ and batch experiments at different pH values. The effect of OS-CS AC on the ability to remove Cr(VI) and the removal mechanism was explored by manipulating the removal conditions, OS-CS AC pore structure, surface chemical properties and applying adsorption isotherms, dynamics, and thermodynamics. The objectives of this study were to (1) investigate the ability of OS-CS AC to remove Cr(VI) and Cr(Total) under different removal conditions; (2) determine the effect of the OS-CS AC pore structure and surface chemistry on the removal of Cr(VI); (3) reveal that the removal of Cr(VI) by OS-CS AC is a spontaneous, endothermic, and chemisorption-based process; and (4) explore the role of electrostatic interactions in the removal of Cr(VI) and the mechanism of Cr(VI) removal by OS-CS AC.

## Materials and methods

### Precursors and reagents

The oily sludge was collected from the reduced tailings of the Tiebiancheng oil sludge station in Changqing, China. It was first dried for 24 h (105 ± 2 ℃) and then subsequently ground and sieved (1.7 mm). The corn stalks were collected from a cornfield in Yan'an City, Shaanxi Province, China and pretreated using a similar method, ground and sieved (0.17 mm). Sodium hydroxide (NaOH), hydrochloric acid (HCl), phosphoric acid (H_2_PO_4_), sulfuric acid (H_2_SO_4_), potassium dichromate (K_2_Cr_2_O_7_), potassium permanganate (KMnO_4_), diphenylcarbazide, and acetone (CH_3_COCH_3_) were purchased from Xi'an Chemical Reagent Factory (Xi'an, China). All chemical agents used in this study were of analytical grade and were used without further purification. The ultrapure water used in the experiments was purchased from Shaanxi Hankang Environmental Protection Technology Company (Xi'an, China).

### Preparation of OS-CS AC

In a N_2_ atmosphere, a mixture of oily sludge and corn stalks in a mass ratio of 1:1 was heated to 650 °C in a pyrolysis furnace and held at this temperature for 1.5 h; the heating rate was 10 °C min^−1^. After the temperature was naturally cooled to room temperature, the obtained raw OS-CS AC was ground to 0.17 mm.

### Batch experiments

A series of batch experiments was conducted to investigate the effects of the initial pH of the solution, OS-CS AC dose, contact time, and temperature on Cr(VI) removal with OS-CS AC. To further study the role of pH in the removal of Cr(VI), the zero charge point of OS-CS AC was determined using the gravimetric method^26^, and a blank control experiment^27^ was used to determine the reason for the change in pH during the removal process. In the experiments, a thermostatic shaker (GTCS-2011, Changzhou Zhengrong Instrument Co., Ltd., Changzhou, China) was used to control the desired temperature. A pH meter (PHS-25 pH meter, China) was used to adjust the pH of the solution. A UV–visible spectrophotometer (Thermo Fisher UV 2350 spectrophotometer, China) was used to measure the absorbance of the filtrate at 540 nm. The shaking rate was maintained at 150 rpm throughout the experiments, and the OS-CS AC dose was 10 g L^−1^. The simulated Cr(VI) wastewater concentration was 50 mg L^−1^.

Batch experiments^28^: OS-CS AC was added to an Erlenmeyer flask filled with 50 mL of Cr(VI) simulated wastewater. The pH of Cr(VI) simulated wastewater was adjusted with 0.1 M HCl and 0.1 M NaOH and measured using a digital pH meter. Then, the Erlenmeyer flasks were agitated in an incubator shaker at the desired rpm and temperature. The solution was passed through a 0.45 μm water filtration membrane with a suction filter device. The Cr(VI) concentration in the residual filtrate after making contact with 1,5-diphenylcarbazide in an acidic environment was determined on a UV-visible spectrophotometer at 540 nm. In addition, the Cr(III) in the filtrate was oxidized to Cr(VI) with 1 M potassium permanganate. After the oxidation of potassium permanganate, the filtrate made contact with 1,5-diphenylcarbazide, and then the total Cr was measured on a 540 nm UV-Vis spectrophotometer. The experiments were repeated independently three times, and the results were reproducible with 5% error. The concentration, Cr(VI) and Cr (total) removal and percentage were determined using the following Eqs. (1–3):

1 $$ {\text{Cr}}\left( {{\text{III}}} \right) = {\text{Cr}}\left( {{\text{total}}} \right) - {\text{Cr}}\left( {{\text{VI}}} \right) $$

2 $${Q}_{\mathrm{e}}=\frac{\left({C}_{0}-{C}_{\mathrm{e}}\right)V}{m}$$

3 $$\eta =\frac{\left({C}_{0}-{C}_{\mathrm{e}}\right)}{{C}_{0}}\times 100\%$$

where *Q*_e_ (mg g^−1^) is the amount of Cr(VI) or total Cr removal, *C*_0_ and *C*_e_ (mg L^−1^) are the initial and e-time concentrations of Cr(VI) and total Cr, respectively, *V* (L) is the volume of the solution, *m* (g) is the weight of OS-CS AC, and $\upeta$ (%) is the removal percentage.

### Adsorption isotherms, kinetics, and thermodynamic models

#### Thermodynamics

Three thermodynamic parameters, Δ*G*, Δ*H*, and Δ*S*, were used to describe the thermodynamics of Cr(VI) removal. Δ*G* was calculated using Eqs. (4–5), while Δ*H* and Δ*S* were obtained by plotting Δ*G* vs. T (Eq. (6))^29^:

4 $$ \Delta G = - RTlnK_{c} $$

5 $${K}_{c}=\frac{{Q}_{e}(m/V)}{{C}_{e}}$$

6 $$ \Delta G = \Delta H - T\Delta S $$

where *R* is the universal gas constant (8.314 J mol^−1^ K^−1^), *T* (K) is the removal temperature, *K*_c_ is the distribution coefficient, *m* (g) is the mass of OS-CS AC, and *V* (L) is the volume of the Cr(VI) solution.

#### Kinetics

To investigate the Cr(VI) removal kinetics, pseudo-first-order kinetics (Lagergren equation) and pseudo-second-order kinetics (McKay equation) were adopted in this study. The nonlinear formulas of these kinetic models are shown below^30,31^:

Lagergren equation

7 $${Q}_{t}={Q}_{e}\left(1-{e}^{-{k}_{1}t}\right)$$

McKay equation

8 $${Q}_{t}=\frac{{k}_{2}{{Q}_{e}}^{2}t}{1+{k}_{2}{Q}_{e}t}$$

where *k*_1_ (min^−1^) and *k*_2_ (min^−1^) are the rate constants of the Lagergren and McKay models, respectively, *Q*_e_ (mg/g) is the saturated removal capacity, and *Q*_t_ (mg/g) is the removal capacity at time *t*.

#### Isotherms

The Langmuir and Freundlich isotherm equations were used to examine the isothermal removal of Cr(VI). The nonlinear formulas of these two equations are as follows^32^:

Langmuir isotherm

9 $${Q}_{e}=\frac{{k}_{L}{Q}_{max}{C}_{e}}{1+{C}_{e}}$$

Freundlich isotherm

10 $${Q}_{e}={k}_{F}{{C}_{e}}^{-n}$$

where *k*_L_ (L mg^−1^) and *k*_F_ are the rate constants of the Langmuir and Freundlich isotherm models, respectively, *Q*_e_ (mg/g) is the equilibrium adsorption capacity, and *C*_e_ (mg L^−1^) is the Cr(VI) concentration when the equilibrium adsorption capacity is reached.

### Characterization

The specific surface area and pore distribution of OS-CS AC were characterized by BET analysis (ASAP-2020-HD88, American Mike Instrument Company), and the data were analysed using Origin2018 software. The crystal structures of the OS-CS AC were characterized using an X-ray polycrystalline diffractometer (D8 Advance, Bruker Instruments, Germany), and the data were collected in the angle range of 10–80 (2*θ*). Scanning electron microscopy (SEM) (EM-30 Plus, Kusem Instrument Company) was used to detect the microstructure and pore distribution of the OS-CS AC. X-ray photoelectron spectroscopy (XPS; EscaLab 250Xi, China) was performed to determine the adsorption of Cr(VI) and Cr(III) on the surface of OS-CS AC, and the data were analysed using Avantage2018 software. An elemental analyser (EAI, German Elemental Analysis System Company) was used to detect the basic properties of OS and CS. The functional groups on the OS-CS AC surface were characterized using a Fourier transform infrared spectrometer (FTIR; Nicolet 5700, Thermo Electron Corporation) and Boehm titration^33^.

### Hydroxy and carboxy masking experiments

The method of masking the carboxyl and hydroxyl functional groups on the surface of OS-CS AC was performed as follows:

OS-CS AC (0.5 g) was added to an Erlenmeyer flask containing absolute ethanol (100 mL) and formaldehyde (100 ml) and immersed for 12 h. Then, the OS-CS AC was filtered, washed repeatedly with ultrapure water to remove excess absolute ethanol or formaldehyde, and dried.

The functional group masking reaction equations are as follows:

11 $$ {\text{RCOOH}} + {\text{CH}}_{{3}} {\text{OH}} + {\text{H}}^{ + } {-\!\!-}{\text{RCOOCH}}_{{3}} + {\text{H}}_{{2}} {\text{O}} $$

12 $$ {\text{2R-OH}} + {\text{HCHO}}{-\!\!-}\left( {\text{R-O}} \right)_{{2}} {\text{CH}}_{{2}} + {\text{H}}_{{2}} {\text{O}} $$

After the reaction, the carboxyl group (–COOH) was converted into an ester group (–COOC–), and the hydroxyl group (R–OH) was converted into an ether group (C–O–C). These –COOH, R–OH, –COOC–, and C–O–C groups can remove Cr(VI) through complexation or ion exchange^34,35^. Therefore, the difference in the ability of OS-CS AC to remove Cr before and after the masking of carboxyl and hydroxyl groups is mainly caused by the difference in the surface charge ability of OS-CS AC, which determines the role of electrostatic interactions in the removal of Cr(VI).

### Desorption-regeneration studies

The OS-CS AC recovered after adsorption equilibrium was reached in the batch experiments was rinsed with distilled water to remove the residual Cr(VI) solution and then dried. The dried OS-CS AC material was placed in a stripping solution contained in a 250 mL Erlenmeyer flask. The solution was 100 mL distilled water adjusted to pH 2 using 0.1 M HCl. The flask was then placed in a thermostatic oscillator for the batch experiments until adsorption equilibrium was reached. The OS-CS AC was recovered, dried and placed in a flask containing 100 mL of a 50 mg/L initial solution of Cr(VI) for the next equilibrium adsorption experiment. This cycle was repeated 3 times. The regeneration performance of OS-CS AC was evaluated by the desorption rate.

13 $$R=\frac{{Q}_{t}}{{Q}_{0}}*100\mathrm{\%}$$

where R is the desorption rate of the OS-CS AC, %; *Q*_*t*_ is the adsorption amount of the composite material after the t-th regeneration, mg/g; and *Q*_*0*_ is the adsorption amount of the carbon material before regeneration, mg/g.

## Results and discussion

### Factors influencing Cr(VI) removal from simulated wastewater

#### pH

**Figure 1:**
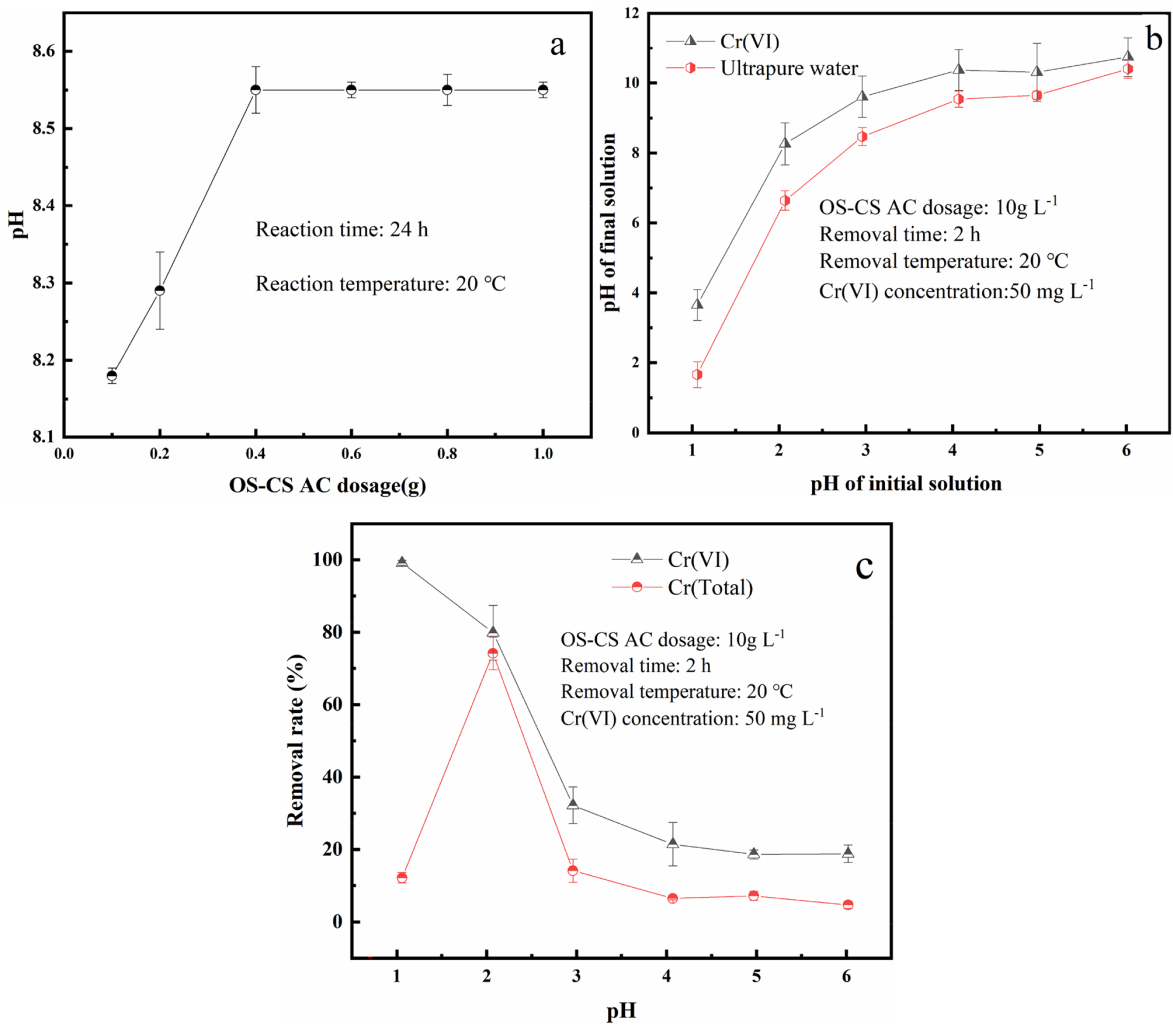
The effect of pH on the surface charge of OS-CS AC (**a**), The effect of OS-CS AC to pH of simulated wastewater and ultrapure water (**b**), The effect of pH to removal rate of Cr(VI) and Cr(Total) (**c**).

Generally, the pH of the solution can directly determine the surface properties of carbon materials and thus affect many chemical reactions^36^. The effect of pH on the surface charge of OS-CS AC is shown in Fig. 1a. With the increase in the quality of OS-CS AC, the pH of the solution stabilized at 8.5; this pH was denoted as the zero charge point (pH_pzc_) of OS-CS AC. The net charge on the OS-CS AC surface was positive when the adsorbate solution was acidic (pH < pH_pzc_). which suggests that under acidic conditions (pH 1–6), the surface chargeability of OS-CS AC is opposite to that of Cr(VI) (CrO_4_^2−^, Cr_2_O_7_^2−^, HCr_2_O_7_^−^), and the electrostatic attraction effect contributes to improving the affinity between the OS-CS AC surface and Cr(VI).

Figure 1b shows the pH change of the solutions when OS-CS AC was added to simulated wastewater and ultrapure water under acidic conditions (pH 1–6). After 2 h of reaction, the pH of the ultrapure water was less than that of the simulated wastewater, indicating that the OS-CS AC consumes a large number of H^+^ ions during Cr(VI) removal. This phenomenon indicates that some Cr(VI) is reduced to Cr(III) during removal. The reduction process can be expressed as follows:

14 $$ {\text{Cr}}_{{2}} {\text{O}}_{{7}}^{{{2} - }} + {\text{14H}}^{ + } + {\text{6e}}^{ - } {-\!\!-}{\text{2Cr}}^{{{3} + }} + {\text{7H}}_{{2}} {\text{O}} $$

15 $$ {\text{HCr}}_{{2}} {\text{O}}_{{7}}^{ - } + {\text{7H}}^{ + } + {\text{3e}}^{ - } {-\!\!-}{\text{Cr}}^{{{3} + }} + {\text{4H}}_{{2}} {\text{O}} $$

16 $$ {\text{Cr}}_{{2}} {\text{O}}_{{7}}^{{{2} - }} + {\text{8H}}^{ + } + {\text{3e}}^{ - } {-\!\!-}{\text{Cr}}^{{{3} + }} + {\text{4H}}_{{2}} {\text{O}} $$

Figure 1c shows the effect of OS-CS AC on the removal of Cr(VI) and Cr(Total) under acidic pH (1–6) conditions. As the pH decreased, the removal capacity of Cr(VI) increased, and the rate of Cr(VI) removal by OS-CS AC increased from 18.79% at pH 6 to 79.86% at pH 2 and then to 99.14% at pH 1. The increase in the Cr(VI) removal rate may be attributed to (1) the increase in the net positive charge on the surface of OS-CS AC at lower pH, resulting in a stronger electrostatic attraction between the surface of OS-CS AC and Cr(VI)^37^, or (2) Cr(VI) has a better ability to be reduced to Cr(III) at lower pH.

From the Cr (total) removal capacity (Fig. 1c), the removal rate of Cr(VI) was always greater than that of Cr (total) during the entire process. This result indicated that the adsorption of Cr(VI) was accompanied by the reduction of Cr(VI) to Cr(III) throughout the removal process. When the pH was increased from 2.0 to 6.0, the rate of total Cr removal decreased. This result may be due to the weakening of the Cr(VI) reduction and adsorption capacity. In addition, the removal of Cr (total) and Cr(VI) were different between pH 1–2. When the pH was increased from 1 to 2, the rate of total Cr removal increased, whereas the removal rate of Cr(VI) decreased. This phenomenon may have occurred because (i) according to Eq. (1), when the pH was 1–2, the reducing ability of Cr(VI) played a major role, and with increasing pH, the reducing ability gradually weakened, resulting in a decrease in the ability to remove Cr(VI); (ii) the cationic charge of Cr(III) was not favourable for binding to the protonated reactive groups of OS-CS AC. With the increase in pH from 1 to 2, the protonation of oxygen-containing functional groups on the surface of OS-CS AC weakened, leading to an increase in the capacity for total Cr removal, and the results were consistent with Mo’s study^38^.

Combining the removal capacity of Cr(VI) and Cr(Total), to explore the effects of the reduction and adsorption of Cr(VI) on the removal, pH 2 was chosen as the follow-up experimental condition.

#### OS-CS AC dose

**Figure 2:**
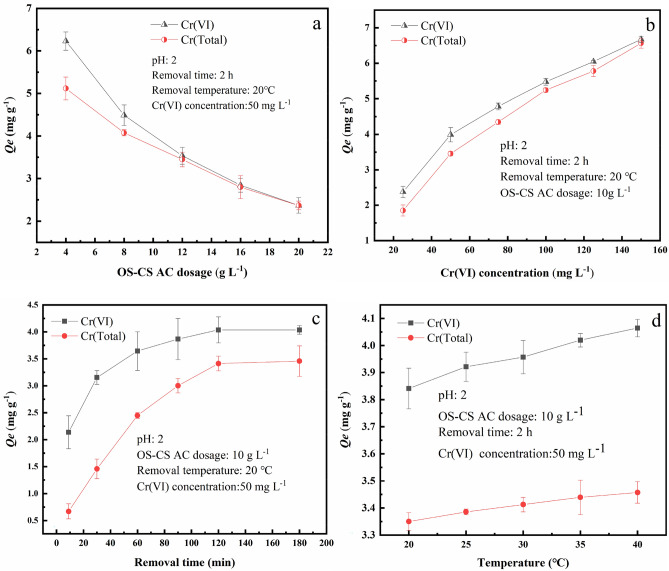
The influence of OS-CS AC dosages on Cr(VI) and Cr(Total) removal (**a**), The influence of the initial concentration of Cr(VI) on the removal of Cr (VI) and Cr (Total) (**b**),The influence of removal time on Cr(VI) and Cr(Total) removal (**c**),The influence of removal temperature on Cr(VI) and Cr(Total) removal (**d**).

The influence of OS-CS AC dose on the removal of Cr(VI) and total Cr removal is shown in Fig. 2a. As the dose of OS-CS AC increased from 4 to 20 g L^−1^, the capacity for Cr(VI) and total Cr removal decreased from 6.23 mg g^−1^ and 5.12 mg g^−1^ to 2.37 mg g^−1^ and 2.36 mg g^−1^, respectively. This decrease may have occurred because more OS-CS AC provides more specific surface area and pore structure and enhances the available sites^39^. Moreover, with the amount of added OS-CS AC changing from 4 to 20 g L^−1^, the amounts of removed Cr(VI) and total Cr gradually became almost the same because enough OS-CS AC provides sufficient electrons for Cr(VI) reduction and sufficient adsorption sites for Cr(III) and Cr(VI).

#### Concentration of Cr(VI)

The effect of the initial concentration of Cr(VI) simulated wastewater on the removal of Cr(VI) and total Cr is shown in Fig. 2b. As the concentration of Cr(VI) was increased, the removal of Cr(VI) and total Cr also increased. This result can be attributed to the fact that by increasing the initial concentration of Cr(VI) simulated wastewater, the effective collisions between OS-CS AC and Cr(VI) increase, which can result in a higher removal capacity of OS-CS AC at higher concentrations of simulated Cr(VI) wastewater^40^.

#### Removal time

As shown in Fig. 2c, removal time had a relatively significant impact on the removal efficiency at the initial time; however, after 30 min, the impact gradually decreased to Cr(VI) removal. When the removal time was between 0 and 30 min, the amount of removed Cr(VI) increased rapidly to 3.15 mg g^−1^, which may be due to the large number of vacant adsorption sites on the surface of the OS-CS AC, which were filled by Cr(VI)^41^. From 30 to 180 min, as the removal time was increased, the removal of Cr(VI) gradually slowed and eventually remained unchanged. This decrease in removal rate may have occurred because the number of binding sites gradually decreased as the removal progressed, slowing the removal rate. In addition, the theoretical removal of Cr(VI) calculated by the McKay equation (Eq. (8)) is close to the amount removed at 120 min; therefore, 120 min was chosen as the final removal time.

#### Removal temperature

Temperature is another significant factor affecting Cr(VI) removal. The effect of temperature on the removal capacity of Cr(VI) and total Cr is shown in Fig. 2d. When the temperature was increased from 20 to 40 °C, the capacity for Cr(VI) removal increased from 3.84 to 4.06 mg g^−1^. In addition, Table 4 shows that Δ*G* decreased as the temperature was increased as the reaction as fit to the Van 't Hoff thermodynamic model, indicating that high temperatures are more conducive to removal. This finding is consistent with previous studies^42^.

### Textural characterizations of OS-CS AC

The well-developed pore structure can provide a good environment for the migration of adsorbates to the internal pores and remove pollutants via pore filling^43^. Since Cr(VI) has a large ion size (1.2 × 0.6 × 0.6 nm^3^), it is necessary to study the influence of the OS-CS AC structure on the removal of Cr(VI). The basic properties of OS and CS are shown in Table 1. The high carbon content and low ash content of corn stalks give OS-CS AC a high potential for a loose and porous structure, and carbon nanotubes may even be formed during the high-temperature pyrolysis process^44^. This demonstrates that the co-pyrolysis of oily sludge and corn stalks offers an alternative for adsorbent preparation.

**Table 1:** Basic features of OS and CS.

Raw material	Moisture content (%)	Volatile content (%)	Ash content (%)	Fixed carbon (%)	N (%)	C (%)	H (%)	S (%)	C/N	C/H
OS	0.89	24.33	67.93	6.85	0.33	12.31	1.33	1.78	37.36	9.26
CS	0.13	96.37	0.58	2.92	1.03	43.53	4.67	0.21	42.42	9.32

**Figure 3:**
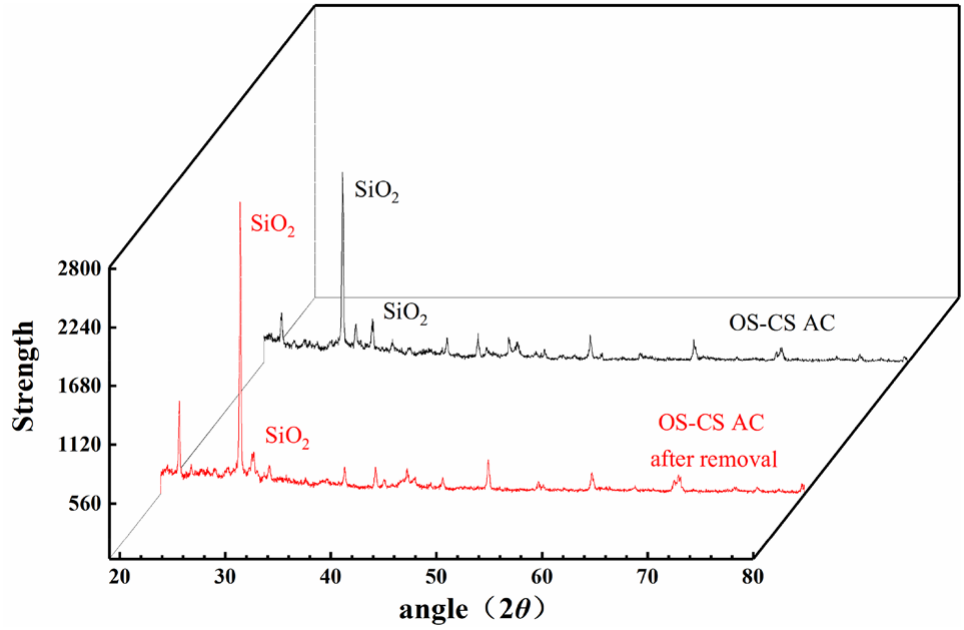
XRD spectra of OS-CS AC (before and after removal of Cr (VI)).

The XRD patterns of the OS-CS AC before and after Cr(VI) removal are shown in Fig. 3. The most significant peak of OS-CS AC before and after removal appears at approximately 2*θ* = 26.20, which indicates that quartz is the main crystal structure of OS-CS AC (before and after removal)^45^.

**Figure 4:**
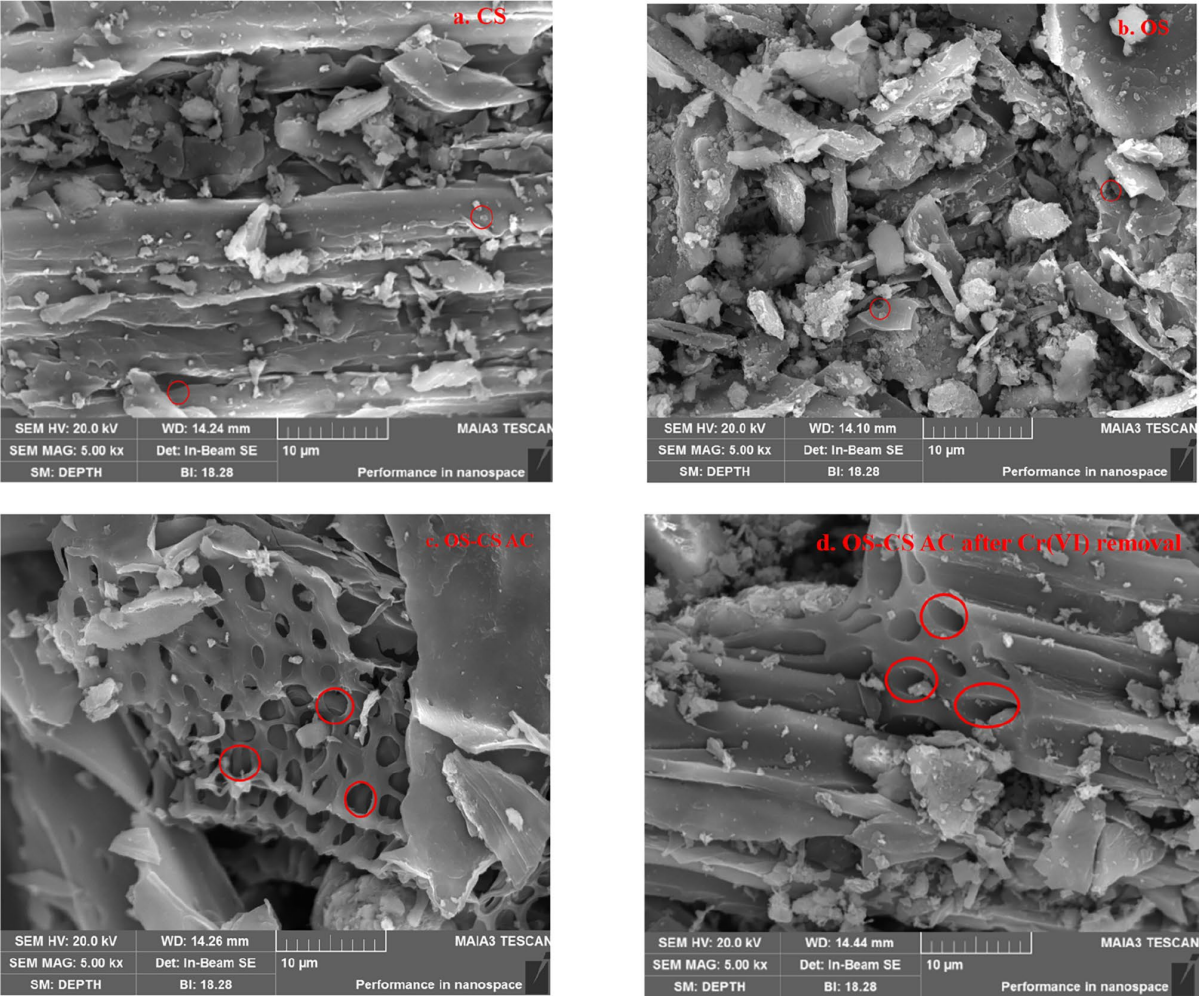
SEM images of CS (**a**), OS (**b**), OS-CS AC (**c**), and OS-CS AC after removal of Cr(VI) (**d**).

Figures 4a–d show SEM images of the raw materials and OS-CS AC (before and after removal), respectively. Comparing Fig. 4a–c, it can be seen that OS-CS AC has a more developed pore structure, possibly because co-pyrolysis destroys the dense structure of the raw materials. In Fig. 4c,d, we can see that after the removal of Cr(VI), the surface of OS-CS AC was relatively weak, and some of the pores were blocked. This result indicates that Cr(VI) binding sites are located in the block and tube of OS-CS AC and between the pores^46^.

The specific surface area, pore volume, and pore size of OS-CS AC (before and after removal) are listed in Table 2. The surface area and pore volume of OS-CS AC were relatively low after the removal of Cr(VI), with a surface area of 21.35 to 19.98 m^2^/g and an average pore size of 17.98 to 13.28 nm, respectively. In addition, the mesopore proportions of OS-CS AC before and after adsorption of Cr(VI) were 98.64% and 92.91%, respectively, indicating that the pore size distribution of OS-CS AC was mainly mesopores.

**Table 2:** Surface area and pore volume of OS-CS AC.

Source	Specific surface area (m^2^/g)	Mesopore average pore size (nm)	Total average pore size (nm)	Mesopore proportion (%)
OS-CS AC	21.35	17.51	17.98	98.64
OS-CS AC (after removal of Cr(VI))	19.98	13.59	13.28	92.91

**Figure 5:**
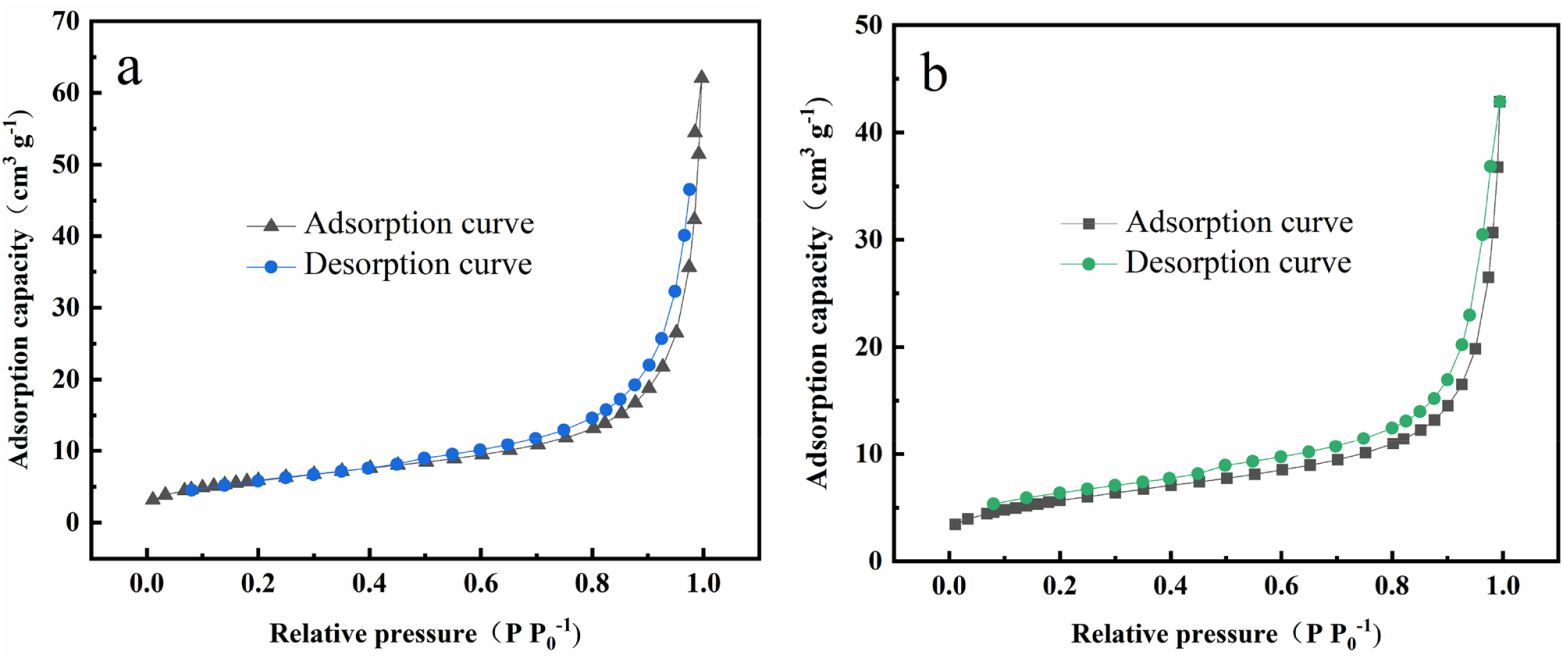
N_2_ adsorption–desorption curves of (**a**) OS-CS AC and (**b**) OS-CS AC after removal of Cr(VI).

Figure 5a,b show the N_2_ adsorption–desorption curves of OS-CS AC before and after Cr(VI) removal, respectively. The N_2_ adsorption–desorption curve of OS-CS AC was closed, and it was classified as a type IV hysteresis loop, which also inedicates that the OS-CS AC had a mostly mesoporous structure, and the curves overlapped at low relative pressures. Then, with increased relative pressures, hysteresis appeared when the relative pressure was 0.45. In addition, after Cr(VI) removal, the OS-CS AC showed hysteresis at the onset of adsorption, which may have occurred because some of the pore structure was consumed by pore filling during Cr(VI) removal.

**Figure 6:**
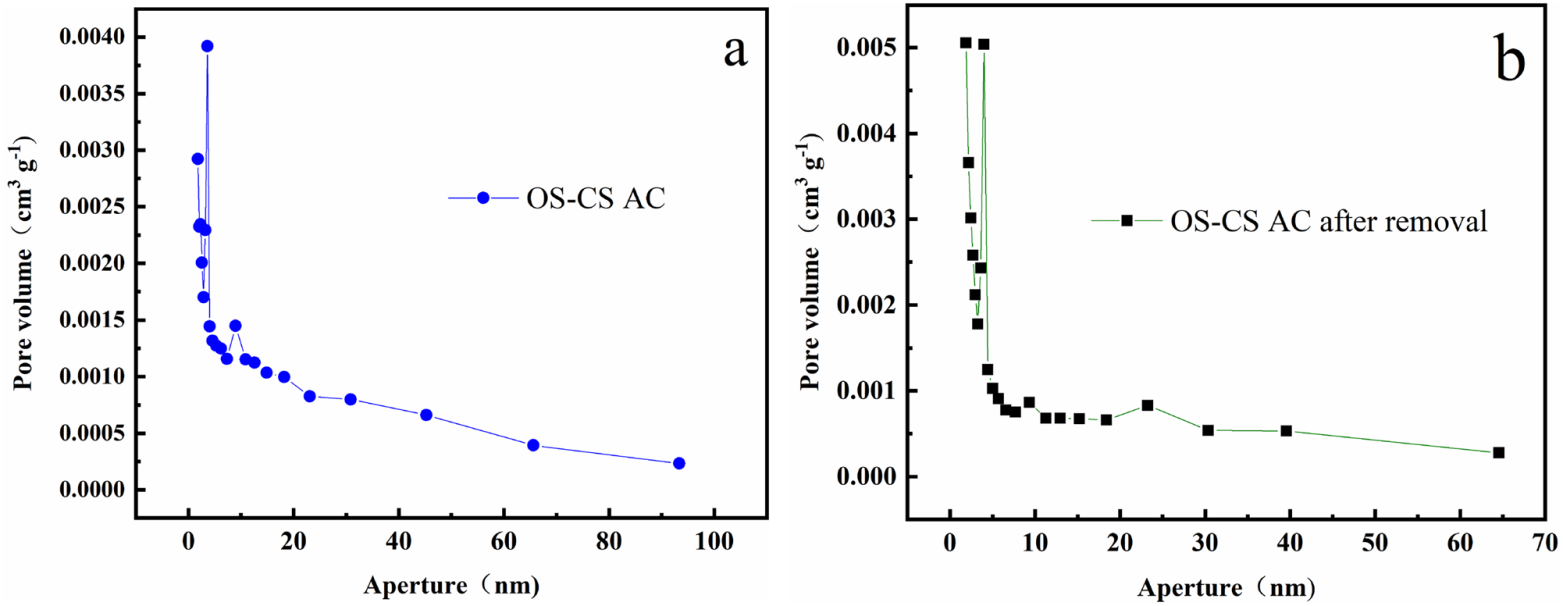
Pore volume curve of (**a**) OS-CS AC and (**b**) OS-CS AC after removal of Cr(VI).

Figures 6a,b show the pore size distribution curves of OS-CS AC before and after Cr(VI) removal, respectively. From Fig. 6a,b, it can be concluded that the pore size distribution of OS-CS AC is relatively concentrated, and the pore structure is dominated by mesopores between 2 and 20 nm. As shown in Fig. 6a with Fig. 6b, the volume of mesopores between 10–20 nm decreased after the removal of Cr(VI), which indicated that the mesopores were more beneficial for transferring Cr(VI), and this result is consistent with those of Sun^47^.

### Chemical surface characterizations of OS-CS AC

**Figure 7:**
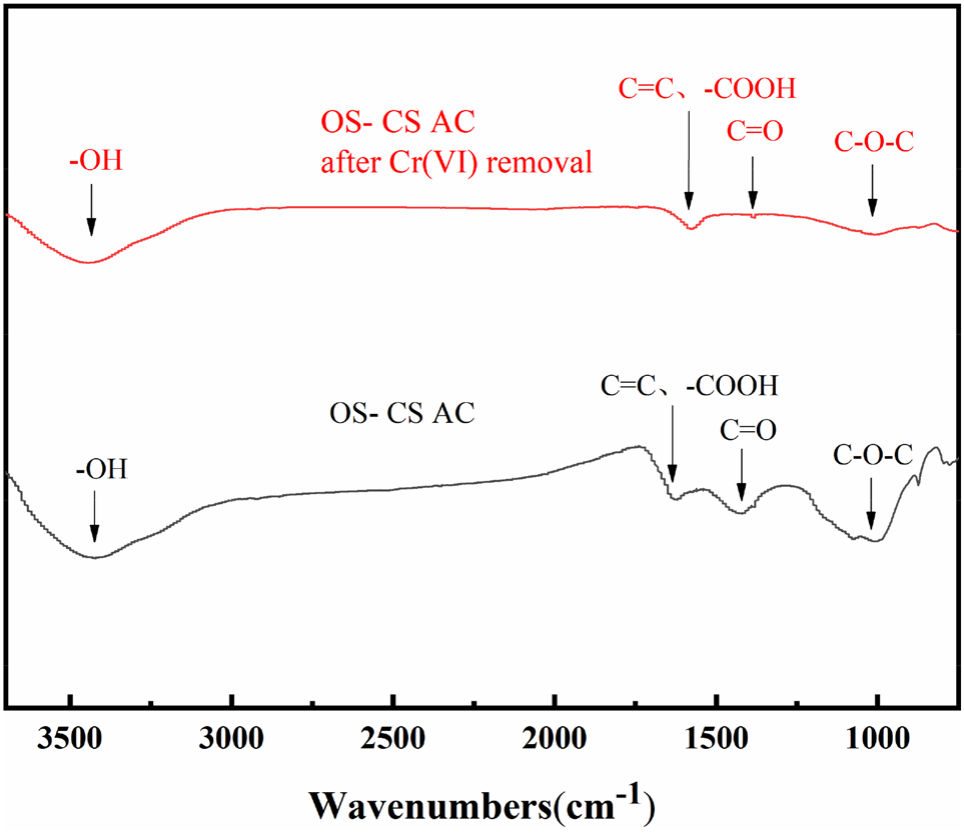
FT-IR spectra of OS-CS AC (before and after removal of Cr (VI)).

The performance of carbon materials is closely related to the pore structure and the chemical properties of the surface^48^. The surface functional groups of OS-CS AC before and after removal were detected by Fourier transform infrared (FTIR) spectroscopy. As shown in Fig. 7, there were stretching vibrations corresponding to –OH groups at 3334 cm^−1^, the vibrations of aromatic C=C and –COOH groups at 1611 cm^−1^, the stretching vibrations of C=O at 1384 cm^−1^, and the tensile vibrations of the C–O–C bond at 1159 cm^−1^^49,50^ in the spectrum of OS-CS AC. However, after removal of Cr(VI), the intensity of the C–O–C peak and C=O weakened, and the absorption peak shifted towards a high wavenumber. This result shows that the C–O–C bond in OS-CS AC was consumed substantially, possibly because the O atom can act as an electron donor and interact with Cr(VI)^51^. In addition, the –COOH absorption peak shifted to a lower wavenumber, and the –OH absorption peak weakened. This result may be due to ionizable –COOH and –OH groups^52^, which can provide H^+^ to participate in the reduction of Cr(VI) (Eqs. (14–16)).

The surface functional group contents of OS-CS AC before and after the removal of Cr(VI) are listed in Table 3. Both acidic and basic functional groups decreased after the removal of Cr(VI). This result indicates that the surface functional groups of OS-CS AC participated in the adsorption process. FT-IR spectroscopy analysis indicates that OS-CS AC has a higher oxygen-containing functional group content and is more conducive to removing Cr(VI).

**Table 3:** Surface functional group content of OS-CS AC.

Source	Carboxyl (mmol/g)	Lactone group (mmol/g)	Phenolic hydroxyl (mmol/g)	Acidity (mmol/g)	Alkalinity (mmol/g)	Total functional group (mmol/g)
OS-CS AC	1.70	0.16	0.48	2.34	3.75	6.09
OS-CS AC (after removal of Cr(VI))	1.32	0.09	0.37	1.78	2.15	3.93

**Figure 8:**
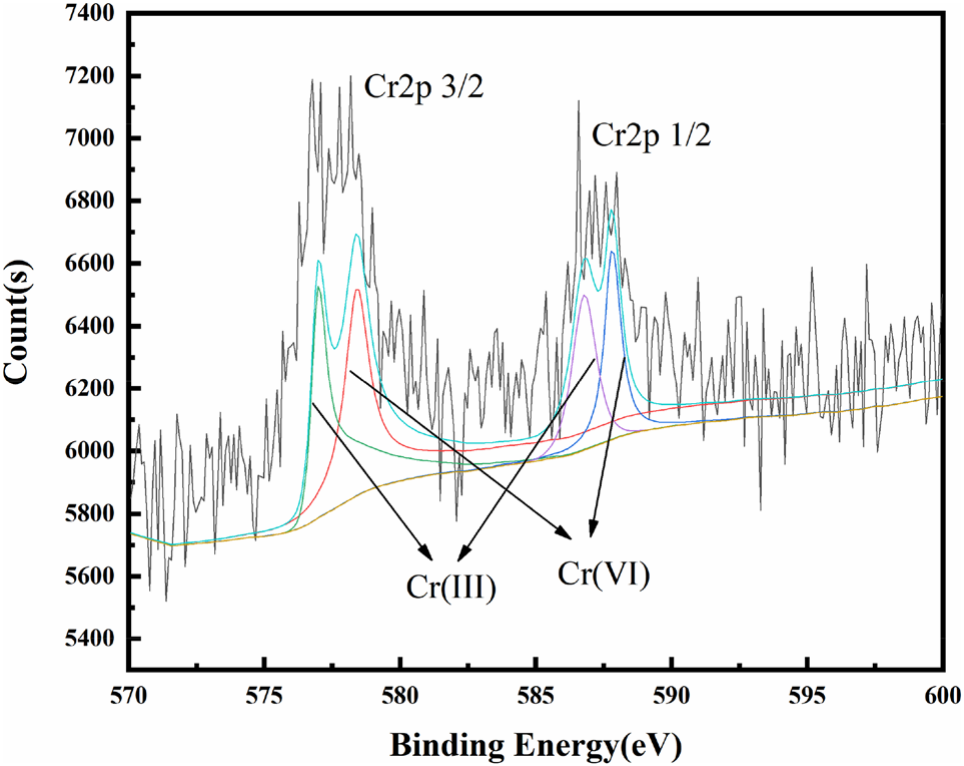
XPS spectrum of OS-CS AC (after removal of Cr(VI)).

The XPS spectrum of Cr 2p after the OS-CS AC removal of Cr(VI) is shown in Fig. 8. The peaks near 587 eV and 577 eV correspond to Cr(VI), while the peaks near 585 eV and 576 eV are characteristic of Cr(III)^53^, which proves that both Cr(III) and Cr(VI) exist on the surface of OS-CS AC after the removal of Cr(VI).

### Characterization of electrostatic interactions

**Figure 9:**
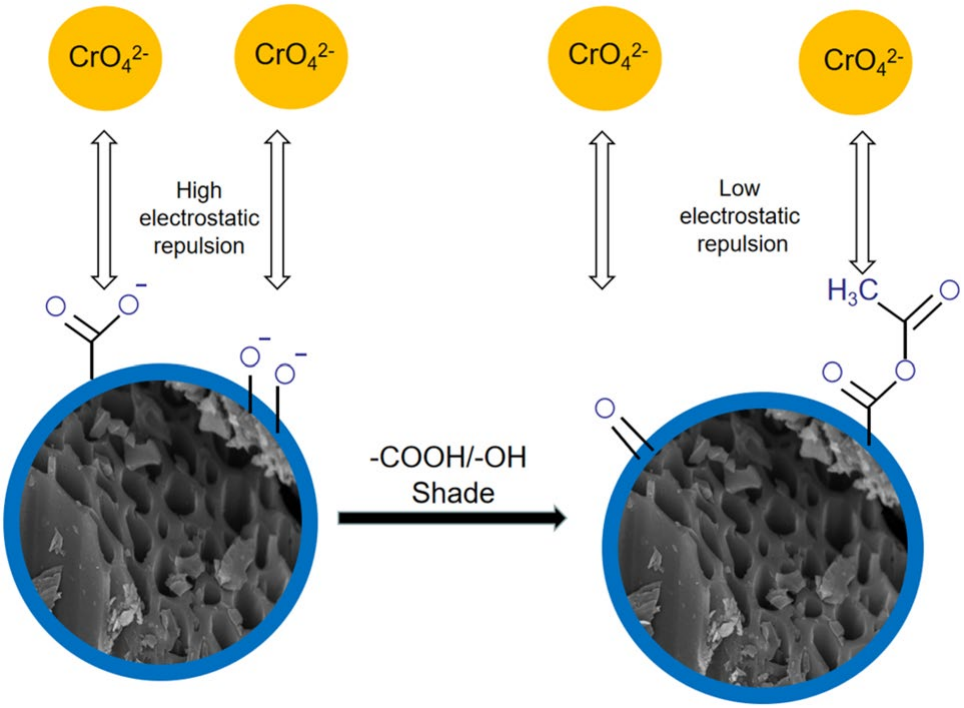
Hydroxy carboxyl group masking mechanism.

**Figure 10:**
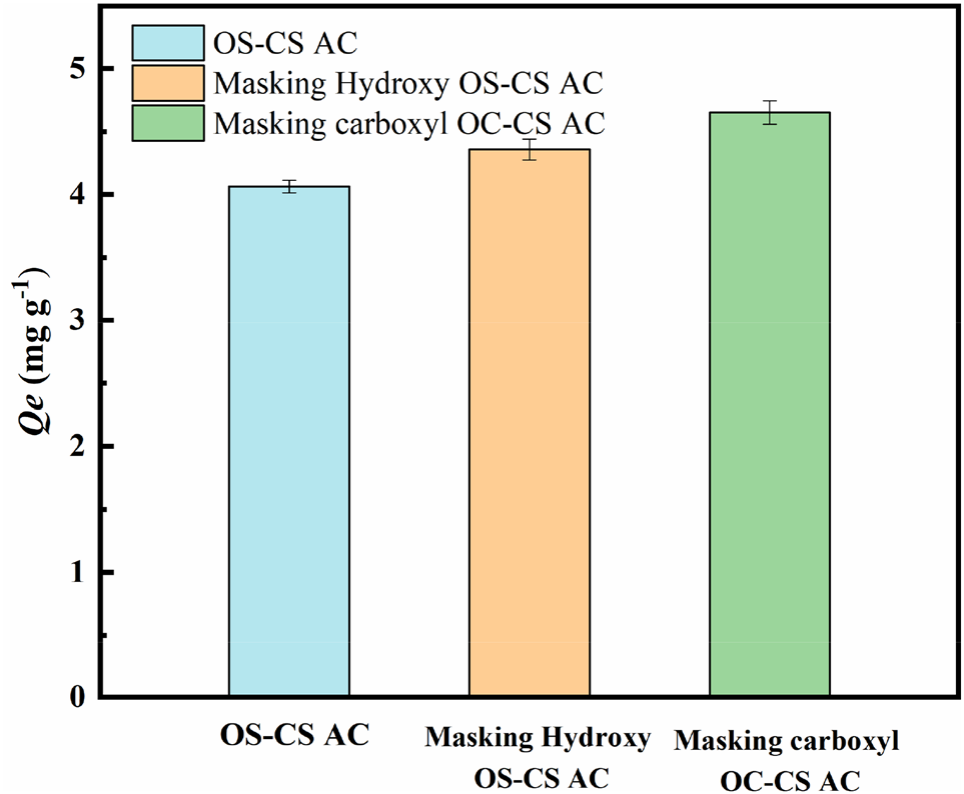
Hydroxyl and carboxyl masking experiment.

The carboxyl and hydroxyl masking experiments (Eqs. (11–12)) converted the carboxyl and hydroxyl groups into ester and ether groups. Both carboxyl and hydroxyl groups can be used as electron donor groups before and after masking^54^. In the mechanism diagram of the masking of the hydroxyl carboxyl group (Fig. 9), the ability to remove Cr(VI) before and after the masking of the carboxyl and hydroxyl groups is mainly due to the different electrostatic interactions at the surface of the OS-CS AC. As shown in Fig. 10, the trend in Cr(VI) removal is as follows: OS-CS AC masking carboxyl group > OS-CS AC masking hydroxyl group > OS-CS AC. This trend may be due to the greater electrostatic attraction between the surface of OS-CS AC and Cr(VI) after the carboxyl and hydroxyl groups were masked and the number of carboxyl functional groups on the surface of the OS-CS AC, which was greater than the number of phenolic hydroxyl functional groups on the OS-CS AC (Table 3). These results indicate that the electrostatic attraction between the electron-donating functional groups of OS-CS AC and Cr(VI) plays an important role in the removal of Cr(VI) by OS-CS AC. Therefore, more positive charges of OS-CS AC lead to larger electrostatic attraction and a greater ability to remove Cr(VI).

### Mechanism of the removal of Cr (VI)

**Figure 11:**
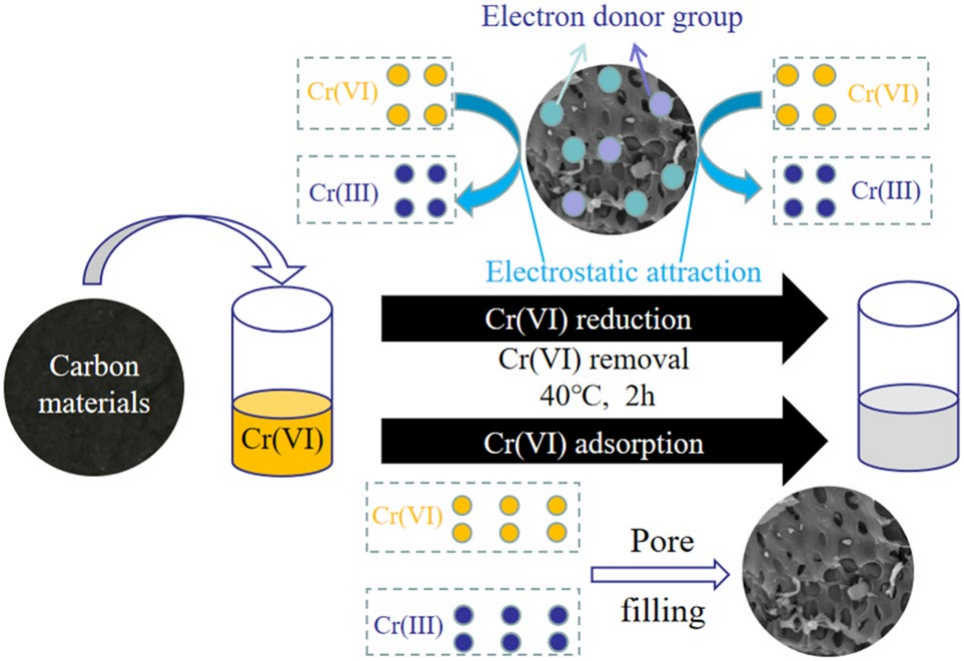
Removal mechanism of Cr(VI) by OS-CS AC.

Based on the electrostatic interactions, the effect of pH and the characterization of the physicochemical properties of OS-CS AC, a possible mechanism for removing Cr(VI) by OS-CS AC is proposed, as shown in Fig. 11. In simulated acidic Cr-containing wastewater, positively charged OS-CS AC (pH_pzc_ = 8.5) and negatively charged Cr(VI) (CrO_4_^2−^, Cr_2_O_7_^2−^, HCr_2_O_7_^−^) were combined under electrostatic attraction. Then, the oxygen-containing functional groups (ether, ketone, carboxyl, and hydroxyl) of the OS-CS AC were used as electron donors^55^, and Cr(VI) accepted these electrons and H^+^, becoming reduced to Cr(III) (Eqs. (14–16)). Based on the reported results, some Cr(III) may be ascribed to the formation of a coordinate covalent bond with the oxygen-containing groups of OS-CS AC and was immobilized in OS-CS AC, and some was released into the aqueous solution due to electrostatic repulsion with OS-CS AC^56^. In addition, Cr(VI) may be due to the filling of pores under the electrostatic attraction of OS-CS AC^57^.

### Thermodynamics, isotherms and kinetics of Cr(VI) removal

**Figure 12:**
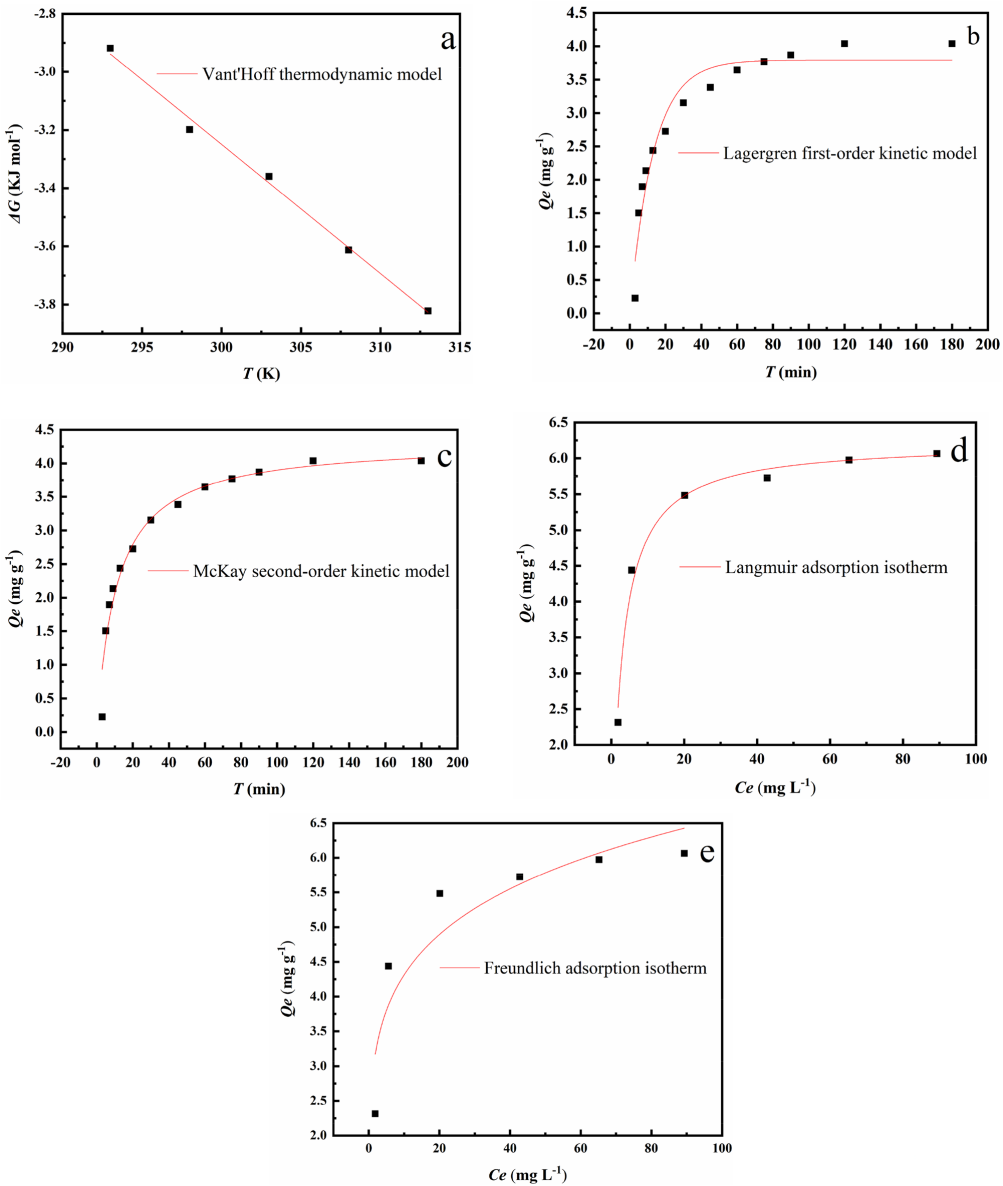
Fitting plots of (**a**) Vant’Hoff thermodynamic, (**b**) Lagergren kinetic, (**c**) McKay kinetic, (**d**) Langmuir adsorption isotherm,and (**e**) Freundlich adsorption isotherm models.

Figure 12a shows the linear fit of the thermodynamic Van't Hoff model. The thermodynamic parameters of the OS-CS AC used to remove Cr(VI) are shown in Table 4. During the removal of Cr(VI) by OS-CS AC, Δ*G*, Δ*H*, and Δ*S* were negative, positive, and positive, respectively, which means that this is a spontaneous, endothermic, and entropy-increasing process. The Δ*S* is likely positive because the OS-CS AC reduced some Cr(VI) to Cr(III) and released it into the aqueous solution, increasing the irregularity and randomness of the solid-aqueous solution interface^58^.

**Table 4:** Summary of linear fitting parameters of thermodynamic model.

Adsorption model	Expression	Correlation coefficient (R^2^)	Fitting parameters	Fitting equation
Vant’Hoff model	Δ*G* = *−RTlnK*_*d*_ ${K}_{d}=\frac{{q}_{e}}{{C}_{e}}$ Δ*G* = Δ*H-T*Δ*S*	0.99	Δ*H* = 10.763 kJ/mol > 0 Δ*S* = 0.0444 kJ/mol > 0 Δ*G* (KJ/mol) < 0	$\Delta G=-0.0444\mathrm{ T}$+10.763

The Lagergren and McKay kinetic fitting curves for Cr(VI) by OS-CS AC are displayed in Fig. 12b,c, and the corresponding parameters are listed in Table 5. From the *R*^2^ value, we can see that the McKay kinetic model is consistent with the experimental results. The Lagergren kinetic model describes the initial stage of Cr(VI) adsorption, while the McKay kinetics describe the entire process of Cr(VI) adsorption, including liquid film diffusion, surface adsorption, and internal diffusion. The adsorption process is accompanied by the formation of chemical bonds^59^. Therefore, it can be inferred that the removal of Cr(VI) by OS-CS AC was mainly due to chemical adsorption.

**Table 5:** Summary of non-linear fitting parameters of kinetic model.

Adsorption model	Lagergren model	McKay model	Langmuir model	Freundlich model
Nonlinear expression	${Q}_{t}={Q}_{e}\left(1-{e}^{-{k}_{1}t}\right)$	${Q}_{t}=\frac{{k}_{2}{{Q}_{e}}^{2}t}{1+{k}_{2}{Q}_{e}t}$	${Q}_{e}=\frac{{k}_{L}{Q}_{max}{C}_{e}}{1+{C}_{e}}$	${Q}_{e}={k}_{F}{{C}_{e}}^{-n}$
Correlation coefficient (R^2^)	0.94	0.96	0.99	0.85
Fit parameters	*Q*_e_ = 3.7942 *k*_1_ = 0.0766	*Q*_e_ = 4.3275 *k*_2_ = 0.0211	*Q*_max_ = 6.2222 *k*_L_ = 0.3668	*n* = − 0.1824 *k*_F_ = 2.8329
Fit equations	${Q}_{t}=3.7942\left(1-{e}^{-0.0766t}\right)$	${Q}_{t}=\frac{0.0211*4.3275t}{1+0.0211*4.3275t}$	${Q}_{e}=\frac{0.3668*6.2222{C}_{e}}{1+{C}_{e}}$	${Q}_{e}=2.8329{{C}_{e}}^{0.1824}$

The Langmuir and Freundlich isotherms of OS-CS AC for the removal of Cr(VI) are shown in Fig. 12d,e. Comparing the Langmuir and Freundlich adsorption isotherm models, we found that the Langmuir adsorption isotherm model better describes the removal of Cr(VI). Therefore, according to the characteristics of the Langmuir adsorption isotherm model, the adsorption sites of OS-CS AC for Cr(VI) were uniformly distributed and had the same affinity, and the adsorption process was mainly chemical adsorption^60^.

### Desorption and regeneration of OS-CS AC

Table 6 shows the rate of Cr(VI) desorption from OS-CS AC at pH 2. The desorption rate changed from 73 to 37% over three sorption cycles, which indicates that it is difficult to reuse OS-CS AC for numerous cycles of sorption and desorption, which may be due to the weak desorption of chromate.

**Table 6:** Desorption results of OS-CS AC.

Sorption cycles	1	2	3
Desorption rate (%)	73	52	37

## Conclusion

From the direct co-pyrolysis of oily sludge and corn stalks, the waste recycling material biochar OS-CS AC was obtained; this material had a Cr(VI) removal rate as high as 99.14%. When the dose of OS-CS AC was 10 g L^−1^ and the concentration of Cr(VI) was 50 mg L^−1^, the removal time and temperature were 2 h and 40 °C, respectively. Greater electrostatic attraction between the electron-donating functional groups of OS-CS AC and Cr(VI) led to a stronger the Cr(VI) removal ability. A greater number of oxygen-containing functional groups on the surface of OS-CS AC improved the ability to reduce Cr(VI) to Cr(III), as this oxygen-containing groups can act as electron donors. In addition, the specific surface area and pore structure of OS-CS AC can affect the adsorption ability for Cr(VI) via pore filling. Moreover, the removal process of Cr(VI) by OS-CS AC better fit the McKay kinetic model (R^2^ > 0.96) and Langmuir isotherm model (R^2^ > 0.99), and the process of Cr(VI) removal was mainly chemical adsorption. Furthermore, the Δ*G*, Δ*H*, and Δ*S* of the Van 't Hoff thermodynamic model were negative, positive, and positive, respectively, indicating that the removal of Cr(VI) was a spontaneous, endothermic, and entropic process. Through this research, we developed an inexpensive, environmentally friendly, and directly obtained adsorbent for the removal of Cr(VI) from wastewater.

## Data Availability

All data generated or analyzed during this study are included in this published article.
